# The association between atherogenic index of plasma, cumulative atherogenic index of plasma and type 2 diabetes mellitus with chronic kidney disease among older Chinese adults: Findings from the CHARLS study

**DOI:** 10.1371/journal.pone.0355200

**Published:** 2026-08-07

**Authors:** Yu Yi, Yiqi Chen, Shulan Feng, Fuhua Lu, La Zhang

**Affiliations:** 1 The Clinical School of Acupuncture Rehabilitation of Guangzhou University of Chinese Medicine, Guangzhou, Guangdong Province, China; 2 The Second Clinical Medical College of Guangzhou University of Chinese Medicine, Guangzhou, Guangdong Province, China; 3 Department of Nephrology, Guangdong Provincial Hospital of Chinese Medicine/the Second Affiliated Hospital of Guangzhou University of Chinese Medicine, Guangzhou, Guangdong Province, China; 4 National Clinical Research Base of Traditional Chinese Medicine for Chronic Kidney Disease, Guangzhou, Guangdong Province, China; 5 State Key Laboratory of Traditional Chinese Medicine Syndrome, The Second Affiliated Hospital of Guangzhou University of Chinese Medicine, Guangzhou, Guangdong Province, China; Barking Havering and Redbridge Hospitals NHS Trust: Barking Havering and Redbridge University Hospitals NHS Trust, UNITED KINGDOM OF GREAT BRITAIN AND NORTHERN IRELAND

## Abstract

**Background:**

The impact of short-term atherogenic burden and long-term cumulative lipid exposure on the risk of incident T2DM with CKD remains unclear. This study examines the longitudinal associations between the Atherogenic Index of Plasma (AIP) and Cumulative Atherogenic Index of Plasma (CumAIP) with the development of T2DM complicated by CKD in middle-aged and older adults.

**Methods:**

A total of 5,779 participants from the CHARLS cohort were included. AIP and CumAIP were used to represent short-term and long-term lipid burdens, respectively. Multivariable logistic regression and restricted cubic spline models were applied to assess the dose-response relationships, adjusting for 11 conventional covariates. ROC curve analysis was conducted to evaluate predictive performance. An exploratory analysis further evaluated incident CKD among participants with T2DM at baseline (n = 494).

**Results:**

Both AIP and CumAIP were significantly associated with incident T2DM with CKD, with the highest quartiles showing modest but statistically significant associations with the outcome (AIP-Q4: OR: 1.013; 95% CI: 1.004–1.022; *p* < 0.05; CumAIP-Q4: OR: 1.017; 95% CI: 1.008–1.026; *p* < 0.001). Linear dose-response associations were observed across logistic regression and RCS regression models. ROC analysis showed that CumAIP demenstrated better discrimination than AIP (AUC 0.683 versus 0.650). In an exploratory analysis of participants with T2DM at the baseline (n = 494), both indices showed suggestive associations with incident CKD (AIP-Q4: OR: 1.050; 95% CI: 1.009–1.145; *p* = 0.025; CumAIP-Q4: OR: 1.081; 95% CI: 1.015–1.152; *p* = 0.016).

**Conclusion:**

AIP and CumAIP is significantly associated with incident T2DM with CKD among Chinese middle-aged and older adults. CumAIP demonstrated superior predictive performance over single AIP measurement. These findings support the potential value of long-term lipid monitoring for identifying indivuduals at increased risk of T2DM with CKD. An exploratory analyses also suggested a similar associations with progression from T2DM to CKD; however, these findings require confirmation in larger cohorts.

## 1. Introduction

The aging population in China faces a growing burden of chronic diseases, with 81.1% of older adults suffering from at least one [1]. Among these, type 2 diabetes mellitus (T2DM) and chronic kidney disease (CKD) are particularly prevalent, with national surveillance reporting rates of 12.4% and 8.2%, respectively [2]. About 40% of individuals with diabetes develop CKD [3], and the comorbidity of the two chronic conditions substantially increases the likelihood of cardiovascular and microangiopathy complications [4]. These findings suggest a potential role of lipid metabolism in the progression of T2DM and CKD by influencing lipid levels.

Previous studies have consistently demonstrated that dyslipidemia is associated with both T2DM and CKD. Abnormal lipid profiles—including elevated triglycerides (TG), total cholesterol (TC), and low-density lipoprotein cholesterol (LDL-C), or reduced high-density lipoprotein cholesterol (HDL-C)—predict diabetes onset [5] and renal outcomes in patients with T2DM [6]. Thus, dyslipidemia may be a key metabolic abnormality linking T2DM with CKD.

The Atherogenic Index of Plasma (AIP), reflecting lipoprotein particle size, has been shown to be associated with atherosclerosis and insulin resistance (IR) [7]. Its cumulative measure, Cumulative Atherogenic Index of Plasma (CumAIP), provides a stable assessment of long-term lipid metabolism. Previous studies have reported a nonlinear association between AIP and both T2DM [8] and CKD [9]. However, the role of AIP and CumAIP in incident T2DM with CKD remains unclear.

The China Health and Retirement Longitudinal Study (CHARLS) offers nationally representative health data on middle-aged and elderly people [10,11]. Using CHARLS, we investigated the correlation between AIP and CumAIP with the risk of new-onset T2DM and CKD (regardless of whether CKD was attributed to diabetes or other causes). Our aim was to clarify the role of dyslipidemia in these two comorbidities.

## 2. Methods

### 2.1. Study population

As a comprehensive nationwide interdisciplinary survey, CHARLS included participants aged 45 and older from 450 villages and communities across 28 provinces (including municipalities and districts). Employing a complex probability sampling method proportional to population, CHARLS includes health-related data from a nationally representative middle-aged and older sample of the Chinese population [12]. Data collection was conducted by trained interviewers using standardized questionnaires to gather extensive information. The CHARLS project has received approval from the Biomedical Ethics Committee at Peking University (IRB00001052–11015), and all participants provided written informed consent. After resting for 15 minutes, the participants had their blood pressure measured in the left arm (averaged over three readings), and their height and weight were measured accurately. Following a 12-hour overnight fast, venous blood samples were collected by trained medical personnel, and immediate complete blood count analyses were performed. All samples were kept at 4 °C and sent to the central laboratory for further testing. The blood glucose, TC, TG, LDL-C, HDL-C and serum creatinine (SCr) levels were then measured.

**Fig 1:**
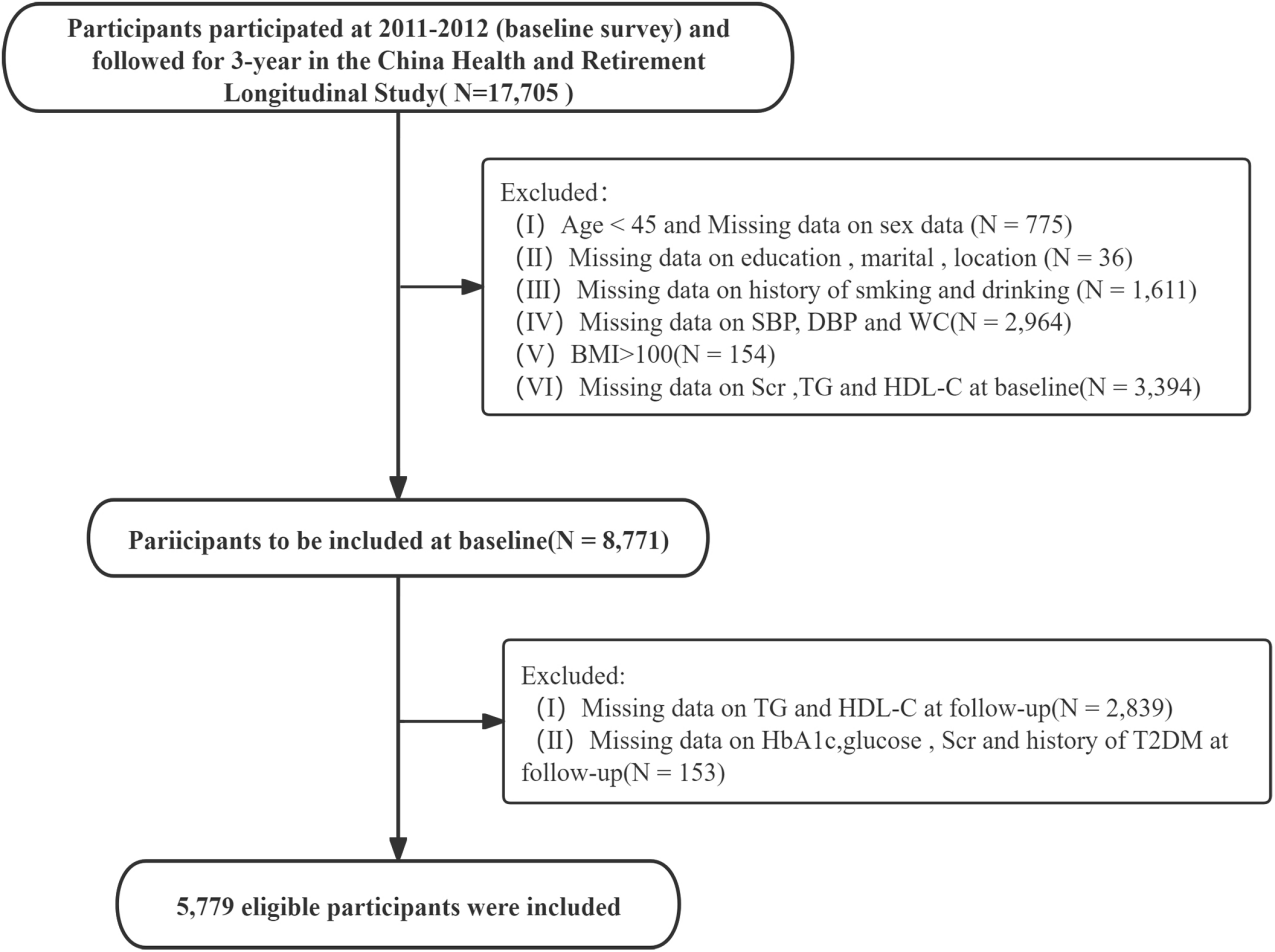
Flow chart of study participants.

In this prospective cohort study, we analyzed longitudinal data from patients with T2DM with CKD (any etiology) sourced from the CHARLS database (http://charls.pku.edu.cn/). We used Wave 1 (2011–2012) as the baseline and Wave 3 (2015–2016) as the end point of the study. The entire research project adhered to the Declaration of Helsinki and the results are reported in accordance with the STROBE Guidelines. We used the following inclusion criteria for the study: age ≥ 45 years, complete sociodemographic information, including sex, education level, marital status, and place of residence. At the baseline, we excluded any participants with missing data on fasting plasma glucose, HbA1c, TG, HDL-C, or SCr. Eventually, 5,779 eligible participants were included (Fig 1).

### 2.2. Research variables

#### 2.2.1. Assessment of AIP and cumulative AIP.

AIP is obtained by measuring the ratio of TG to HDL-C in the blood, followed by calculating the logarithm of this ratio. CumAIP refers to the cumulative effect of long-term trend analysis of an individual’s AIP values over a period. Its value is calculated as the ratio of the sum of the AIP values measured during the first and third assessment waves to the total exposure duration.

AIP calculation [13]:


$$\begin{array}{c} \text{AIP }=\text{ log10 }[\text{TG }(\text{mg}/\text{dL})/\text{HDL}-\text{C}(\text{mg}/\text{dL})] \end{array}$$

CumAIP calculation [14]:


$$\begin{array}{c} \left({\text{AIP}}_{\text{2012}}+{\text{AIP}}_{\text{2015}})/\text{2}\times (\text{2015}-\text{2012}\right) \end{array}$$

#### 2.2.2. Assessment of type 2 diabetes mellitus with chronic kidney disease.

Based on criteria published by the American Diabetes Association in 2005 [15], diabetes in this study was defined as fasting blood glucose levels ≥126 mg/dl (7 mmol/L), and/or random blood glucose levels ≥ 200 mg/dl (11.1 mmol/L), and/or HbA1c levels ≥6.5%, and/or a self-reported diagnosis of “yes” (“ Have you ever been diagnosed with diabetes or hyperglycemia?).

Because repeated eGFR measurements and markers of kidney damage (e.g., proteinuria) were unavailable in the CHARLS database, CKD was operationally defined as an eGFR < 60 mL/min/1.73 m^2^ at follow-up. Accordingly, the definition of CKD in this study should be interpreted as an operational approximation rather than a fully KDIGO guidelines-compliant definition. The eGFR was calculated using the CKD-EPI equation [16], with sex-specific formulas based on SCr (mg/dL) thresholds:

For males:

If SCr ≤ 0.9: 141×(SCr/0.9) ^-0.411^×(0.993)^age^

If SCr > 0.9: 141×(SCr/0.9)^-1.209^×(0.993)^age^

For females:

If SCr ≤ 0.7: 144×(SCr/0.7)^-0.392^×(0.993)^age^

If SCr > 0.7: 144×(SCr/0.7)^-1.209^×(0.993)^age^

#### 2.2.3. Assessment of covariates.

We modified the analyses to account for sociodemographic characteristics, health-related behaviors, and anthropometry. As demographic variables, we analyzed age, sex, education (primary school and below, high school and junior college and above), location (rural or urban), and marital status (married or other). In addition, we examined smoking status (current smoker or non-smoker) and alcohol consumption (current drinker or non-drinker) as variables for health-related behaviors. These data were obtained through self-reported questionnaires with the help of trained interviewers. Anthropometric data included SBP, DBP, BMI, and waist circumference (WC). BMI was calculated using the following formula: weight/height^2^ (kg/m^2^).

### 2.3. Data analysis

For descriptive data, continuous variables with a normal distribution are expressed as means and standard deviations (SD), while continuous variables with a non-normal distribution are expressed as median with interquartile range (IQR). Categorical variables are presented as percentages. Univariate analysis of variance, the Mann-Whitney U test, or the chi-square test were utilized to compare baseline characteristics and the incidence of T2DM with CKD in the AIP quartiles (Q1, Q2, Q3, and Q4). We employed three logistic regression models to investigate the associations between AIP, CumAIP, and T2DM with concurrent CKD (any etiology). These models included: a crude model without adjustments (Model 1), a model adjusted for age, sex, educational level, residential location, and marital status (Model 2), and a fully adjusted model that additionally incorporated smoking status, alcohol consumption, SBP, DBP, BMI and WC (Model 3). All results were presented as odds ratios (ORs) with corresponding 95% confidence intervals (CIs). The predictive ability of AIP/CumAIP for T2DM with comorbid CKD was evaluated using the area under the receiver operating characteristic (ROC) curve. Furthermore, prior to covariate adjustment, we conducted logistic linear regression analysis to explore the linear relationship between exposure and outcome variables. After adjusting for covariates, we employed restricted cubic splines (RCS) to examine potential nonlinear associations and to visualize the dose-response relationships between both AIP and CumAIP with T2DM with concurrent CKD. Finally, we performed interaction using product terms (AIP/CumAIP ×[interaction terms]) to determine whether the associations between AIP/CumAIP and the outcomes had been modified by sociodemographic characteristics, health-related behaviors, or anthropometric measures.

All statistical analyses were performed using R (version 4.4.1). The restricted cubic splines were implemented using the “rms” package, while the optimal cutoff points were determined using the “CatPredi” package. A two-tailed *p*-value < 0.05 was considered statistically significant.

## 3. Results

### 3.1. Baseline characteristics

For the primary analysis, a total of 5,779 participants without T2DM and CKD at baseline were included (median age 58 years; 55.6% female), of whom 77 had developed new-oneset T2DM with CKD during follow-up. These individuals were older and had higher BMI, WC, and SBP levels, along with higher proportions of alcohol consumption and smoking compared with those without comorbidities (Table 1).

**Table 1:** Participant characteristics at baseline (N = 5,779).

	Level	Total	Not diseased	T2DM With CKD	*P*
Age, years		58 (52,64)	58 (52,64)	66 (60,71)	<0.001
Sex, n (%)					0.124
	Female	3,215 (55.6)	3,165 (55.5)	50 (64.9)	
	Male	2,564 (44.4)	2,537 (44.5)	27 (35.1)	
Marital status, n (%)					0.146
	Married	5,139 (88.9)	5,075 (89.0)	64 (83.1)	
	Non-married	640 (11.1)	627 (11.0)	13 (16.9)	
Education, n (%)					0.007
	College or above	146 (2.5)	144 (2.5)	2 (2.6)	
	High school	1,498 (25.9)	1,490 (26.1)	8 (10.4)	
	Primary school or below	4,135 (71.6)	4,068 (71.3)	67 (87.0)	
Location, n (%)					0.03
	City or town	878 (15.2)	859 (15.1)	19 (24.7)	
	Village	4,901 (84.8)	4,843 (84.9)	58 (75.3)	
Drinking, n (%)					0.001
	Drink, but less than once a month	476 (62.7)	474 (8.3)	2 (2.6)	
	Drink more than once a month	1,196 (20.7)	1,191 (20.9)	5 (6.5)	
	None of these	4,107 (71.1)	4,037 (70.8)	70 (90.9)	
Smoking, n (%)					0.013
	Current smoker	1,677 (29.0)	1,666 (29.2)	11 (14.3)	
	Ex-smoker	476 (8.2)	470 (8.2)	6 (7.8)	
	Non-smoker	3,626 (62.7)	3,566 (62.5)	60 (77.9)	
BMI, kg/m^2^		23.28 (21.05, 25.95)	23.24 (21.03, 25.92)	24.96 (23.46, 28.77)	<0.001
WC, cm		85.0 (78.0, 92.2)	85.0 (78.0, 92.0)	92.5 (83.0, 100.2)	0.003
SBP, mmHg		127.00 (114.67, 142.00)	127.00 (114.33, 141.67)	139.00 (126.67, 151.67)	<0.001
DBP, mmHg		74.67 (67.33, 83.00)	74.67 (67.33, 83.00)	75.67 (69.00, 87.00)	0.231
AIP		−0.04 (−0.25, 0.19)	−0.04 (−0.25, 0.18)	0.15 (−0.07,0.38)	<0.001
Quartiles of AIP					<0.001
	Q1	1,445 (25.0)	1,438 (25.2)	7 (9.1)	
	Q2	1,445 (25.0)	1,429 (25.1)	16 (20.8)	
	Q3	1,444 (25.0)	1,424 (25.0)	20 (26.0)	
	Q4	1,445 (25.0)	1,411 (24.7)	34 (44.2)	
AIP per IQR		−0.09 (−0.57, 0.43)	−0.09 (−0.57, 0.42)	0.35 (−0.15, 0.88)	<0.001
Cumulative AIP		−0.06 (−0.58, 0.51)	−0.06 (−0.58, 0.50)	0.49 (−0.09, 1.11)	<0.001
Quartiles of Cumulative AIP					<0.001
	Q1	1,445 (25.0)	1,439 (25.2)	6 (7.8)	
	Q2	1,445 (25.0)	1,429 (25.1)	16 (20.8)	
	Q3	1,444 (25.0)	1,427 (25.0)	17 (22.1)	
	Q4	1,445 (25.0)	1,407 (24.7)	38 (49.4)	
Cumulative AIP per IQR		−0.06 (−0.53, 0.47)	−0.06 (−0.53, 0.46)	0.45 (−0.08, 1.02)	<0.001

Abbreviations: BMI: body mass index; WC: waist circumference; SBP: systolic blood pressure; DBP: diastolic blood pressure; AIP: atherogenic index of plasma; cumulative AIP: cumulative atherogenic index of plasma

In the exploratory analysis of participants with baseline T2DM (n = 494), only 31 (6.3%) had progressed to CKD. Descriptive comparisons suggested that higher baseline AIP and older age were associated with progression (S2 Table), but formal inference was limited by the small number of events.

### 3.2. The dose-response relationship between AIP and T2DM with CKD

#### 3.2.1. Primary analysis: Incident T2DM with CKD in the overall cohort.

Multivariable analyses revealed a significant dose-response relationship between AIP levels and T2DM with CKD (*p* < 0.001). After full covariate adjustment, the participants in the highest AIP quartile (Q4) had modestly higher odds of the outcome compared with those in the lowest quartile (Q1) (OR: 1.013, 95% CI: 1.004–1.022, *p* < 0.01) (Table 2). While the effect size per IQR increase was small, the dose-response trend across quartiles remained statistically robust after multivariable adjustment (*p* for trend = 0.005).

**Table 2:** AIP’s association with the risk of T2DM with CKD in the CHARLS.

	Model 1	*p*	Model 2	*p*	Model 3	*p*
AIP per IQR	1.010 [1.006, 1.014]	<0.001	1.010 [1.006, 1.014]	<0.001	1.007 [1.003, 1.011]	<0.001
Quartiles of AIP						
Q1	Ref		Ref		Ref	
Q2	1.006 [0.998, 1.015]	0.144	1.006 [0.998, 1.015]	0.140	1.004 [0.996, 1.013]	0.337
Q3	1.009 [1.001, 1.018]	0.035	1.009 [1.001, 1.017]	0.034	1.004 [0.996, 1.013]	0.316
Q4	1.019 [1.010, 1.027]	<0.001	1.019 [1.011, 1.028]	<0.001	1.013 [1.004, 1.022]	0.004
*P* for trend		<0.001		<0.001		0.005

Notes: Model 1 represents the crude model. Model 2 was adjusted for age, sex, educational attainment, region of residence, and marital status. Model 3 was further adjusted for BMI, SBP, DBP, WC, smoking status and alcohol consumption. AIP per IQR represents the odds ratio associated with an increase of 0.44 units in the AIP, corresponding with the interquartile range observed in this cohort (Q1 = −0.25, Q3 = 0.19).

#### 3.2.2. Exploratory ananlysis: Incident CKD in participants with baseline T2DM.

In the exploratory analysis of the baseline T2DM subgroup, similar directional associations were observed, although the wide confidence intervals and small event number warrant cautious interpretation (OR: 1.050, 95% CI: 1.009–1.145, *p* = 0.025) (S3 Table).

### 3.3. The dose-response relationship between CumAIP and T2DM with CKD

#### 3.3.1. Primary analysis: Incident T2DM with CKD in the overall cohort.

CumAIP showed a significant dose-dependent association with T2DM with CKD (*p* < 0.001). Comparing with Q1, participants in the highest CumAIP quartile (Q4) had a higher risk (adjusted OR: 1.017, 95% CI: 1.008–1.026, *p* < 0.001) (Table 3).

**Table 3:** CumAIP’s association with the risk of T2DM with CKD in the CHARLS.

	Model 1	*p*	Model 2	*p*	Model 3	*p*
Cumulative AIP per IQR	1.012 [1.008, 1.016]	<0.001	1.012 [1.009, 1.016]	<0.001	1.010 [1.005, 1.014]	<0.001
Quartiles of CumAIP						
Q1	Ref		Ref		Ref	
Q2	1.007 [0.999, 1.010]	0.104	1.007 [0.999, 1.016]	0.091	1.004 [0.996, 1.013]	0.294
Q3	1.008 [0.999, 1.016]	0.073	1.008 [0.999, 1.017]	0.053	1.003 [0.995, 1.012]	0.427
Q4	1.022 [1.014, 1.031]	<0.001	1.024 [1.015, 1.032]	<0.001	1.017 [1.008, 1.026]	<0.001
*P* for trend		<0.001		<0.001		<0.001

Notes: Model 1 represents the crude model. Model 2 was adjusted for age, sex, educational attainment, region of residence, and marital status. Model 3 was further adjusted for BMI, SBP, DBP, WC, smoking status and alcohol consumption. Cumulative AIP per IQR represents the odds ratio associated with an increase of 1.09 units, corresponding to the interquartile range of cumulative AIP in this cohort (Q1 = −0.58, Q3 = 0.51).

#### 3.3.2. Exploratory ananlysis: Incident CKD in participants with baseline T2DM.

In the exploratory analysis restricted to participants with baseline T2DM (n = 494), a similar association was confirmed in the T2DM-to-CKD progression subgroup (OR: 1.081, 95% CI: 1.015-1.152; *p* < 0.05) (S4 Table). Due to the small subgroup size and limited number of events, this exploratory analysis carries substantial uncertainty.These findings should be considered hypothesis-generating and require validation in larger cohorts.

### 3.4. RCS analysis of AIP/CumAIP and T2DM with CKD

**Fig 2:**
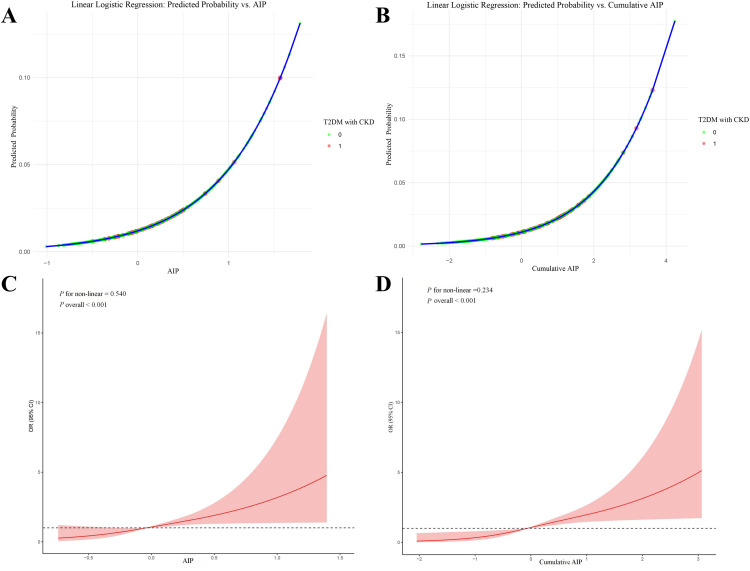
Logistic linear regression model and RCS curve analysis results. (A) Logistic linear regression model of the association between CumAIP and the risk of T2DM with CKD; (B) Logistic linear regression model of the association between CumAIP and the risk of T2DM with CKD; (C) RCS curves of the association between AIP and the risk of T2DM with CKD; (D) RCS curves of the association between CumAIP and the risk of T2DM with CKD.

Restricted cubic spline analysis for the primary outcome showed a significant linear positive association between both AIP and CumAIP and the risk of T2DM with CKD (Fig 2). These associations persisted after covariate adjustment, and when the AIP/CumAIP values were positive, the entire confidence interval for the risk estimates remained above an odds ratio of 1.

### 3.5. ROC curve analysis of AIP/CumAIP for predicting T2DM with CKD

**Fig 3:**
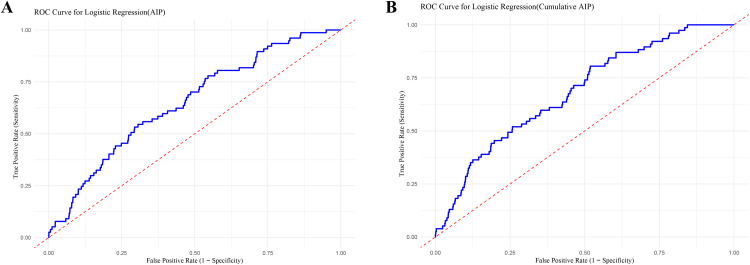
ROC Curve analysis results. (A) ROC curve analysis for predicting the association between AIP and the risk of T2DM with CKD; (B) ROC curve analysis for predicting the association between CumAIP and the risk of T2DM with CKD.

The AIP model yielded an AUC of 0.650 (95% CI: 0.623–0.678) (Fig 3) with an optimal cutoff value of 0.517. The CumAIP model showed better discrimination (AUC = 0.683, 95% CI: 0.651–0.715), and dichotomization further improved its classification accuracy (AUC = 0.698, 95% CI: 0.669–0.727) (Fig 3). Overall, the CumAIP model demonstrated superior predictive ability compared with the AIP model.

### 3.6. Stratified analysis

**Fig 4:**
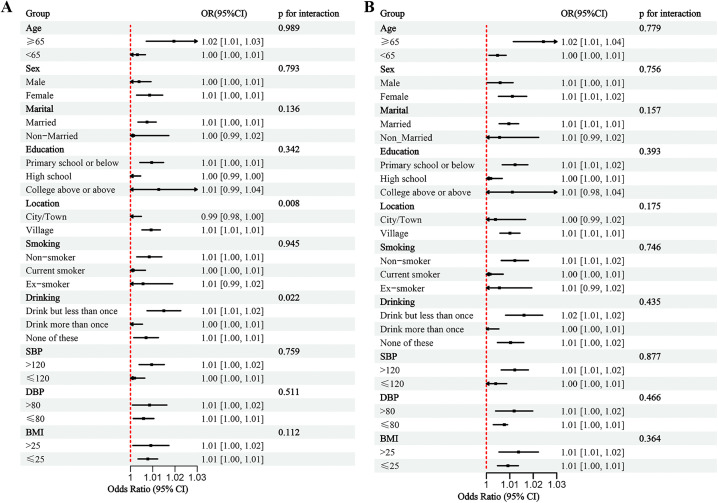
Forest plot of stratified analysis results. (A) Forest plot of stratified analysis of the association of AIP with the risk of T2DM with CKD; (B) Forest plot of stratified analysis of the association of CumAIP with the risk of T2DM with CKD.

Stratified analysis showed that the association between CumAIP and T2DM with CKD was consistent across all subgroups. By contrast, AIP displayed significant interaction with drinking status and geographic region (*p* < 0.05) (Fig. 4).

## 4. Discussion

In the nationally representative CHARLS cohort, both AIP and CumAIP were positively associated with the risk of T2DM with CKD. Moreover, CumAIP showed superior predictive performance compared with a single baseline AIP measure. These findings suggest that long-term lipid exposure is associated with the development of these conditions.

Previous studies have highlighted the heterogeneity of CKD among patients with T2DM, including diabetic kidney disease (DKD), non-diabetic kidney disease (NDKD), and their coexistence [17,18]. Renal impairment in diabetes often involves multifactorial mechanisms and cannot be fully explained by DKD or proteinuria alone [19]. Our findings extend this evidence by demonstrating that both AIP and CumAIP, as lipid-related indices, are independently associated with the risk of T2DM with CKD. These associations suggest that dyslipidemia may serve as a marker of an additional pathway associated with disease progression. As the patient population with cardiorenal syndrome expands, it has been observed that there may be common influencing factors for damage to the heart and kidneys, including indicators of dyslipidemia. Several biological mechanisms may explain the observed associations. Previous studies have shown that higher AIP is linearly related to T2DM mortality [20], while CumAIP reflects the cumulative impact of lipid abnormalities and inflammation on diabetic progression [21]. Dyslipidemia can impair β-cell function though cholesterol accumulation and defective insulin secretion [22], and lower HDL-C further promotes β-cell apoptosis via endoplasmic reticulum stress [23]. In addition, abnormal lipid profiles, including elevated TG and reduced HDL-C, have been linked to increased CKD risk [24]. Oh et al. [25] found a significant association between AIP and renal outcomes in patients with metabolic dysfunction-associated disorders. Moreover, cholesterol deposition in podocytes may induce cellular stress and apoptosis, ultimately contributing to renal dysfunction [26]. Additionally, clinical investigations of obicetrapib have demonstrated that this pharmacological agent not only modulates lipid profiles, but also attenuates the advancement of renal impairment, indicating a potentially significant role of lipids in the pathogenesis of CKD [27]. Together, these findings suggest that dyslipidemia is associated with a shared pathway related to both T2DM and CKD, which is consistent with the predictive value of AIP and CumAIP observed in our study.

At the molecular level, additional mechanisms have been proposed to link lipid metabolism with diabetes and kidney disease. AIP has been considered a surrogate marker of IR [8]. Also, elevated TG and free fatty acids promote excessive reactive oxygen species (ROS) production and inflammatory mediator release, impairing insulin signaling and β-cell function [28]. Dysregulated lipid metabolism also induces mitochondrial fission and oxidative stress, leading to renal tubular epithelial cell apoptosis and glomerular barrier injury [29]. In adipose tissue, abnormal lipid turnover activated microinflammation and lipid peroxidation cascades, further aggravating systemic insulin resistance [30]. Moreover, decreased HDL-C impairs reverse cholesterol transport, increasing the proportion of small, dense lipoproteins that accumulate in glomeruli and renal tubules, thereby accelerating kidney injury [25]. These molecular insights provide biological plausibility for the epidemiological associations between AIP/CumAIP and T2DM with CKD.

It is important to recognize that the association between dyslipidemia and these two diseases is likely bidirectional. In addition to the potential influence of lipid abnormalities on disease progression, T2DM can promote dyslipidemia through disturbances in lipoprotein metabolism—including elevated very-low-density lipoprotein (VLDL) levels, hypertriglyceridemia, and reduced HDL-C. Moreover, CKD may contribute to secondary dyslipidemia by diminishing lipolytic enzyme activity and disrupting apolipoprotein synthesis and catabolism. However, this is a cohort study, which can only demonstrate the correlation between variables, but cannot establish a causal relationship. Although statistically significant, the observed associations were modest in magnitude. This likely reflects the multifactorial etiology of T2DM and CKD, wherein dyslipidemia represents one contributory pathway, rather than a dominant determinant. Additionally, the narrow IQR of AIP in this cohort (0.44 units) may have constrained the observable effect size. Importantly, CumAIP demonstrated discrimination superior to that of the single-point AIP (AUC 0.683 vs. 0.650), suggesting that cumulative lipid burden may provide greater clinical utility than isolated measurements.

This study has several strengths: Firstly, it is the first to evaluate both AIP and CumAIP in relation to the incident T2DM with CKD in a large, nationally representative Chinese cohort. Secondly, we incorporated dose-response analyses and developed a predictive model for both indices. The CumAIP-based model demonstrated superior discrimination compared with baseline AIP, highlighting its potential utility in clinical risk stratification. Thirdly, the use of CHARLS data, with its standardized design and extensive follow-up, enhances the reliability and generalizability of our findings within the Chinese population.

Several limitations should also be noted. Firstly, the study population was restricted to China, which may limit extrapolation to other ethnic groups. In addition, although AIP and CumAIP were identified as significant risk factors, the underlying molecular mechanisms linking lipid abnormalities to T2DM and CKD require further investigation. Moreover, the small number of incident T2DM-CKD cases and the lack of key renal biomarkers, such as urinary protein, may have led to underestimation of disease prevalence, particularly in early stages. Due to the unavailability of data regarding urinary protein levels, this study’s findings are limited to patients exhibiting confirmed renal dysfunction. Therefore, our findings may have led to underdiagnosis of patients with proteinuria but normal renal function, in the early stages of chronic kidney disease. Future large-scale, multiethnic cohorts with more comprehensive laboratory data are needed to validate and extend these findings.

## 5. Conclusion

Using nationally representative data from the CHARLS cohort, this study revealed that both AIP and CumAIP are significantly associated with the incident T2DM with CKD among Chinese middle-aged and older adults. CumAIP showed better predictive performance than AIP, highlighting the value of long-term lipid monitoring for risk statification. Given that the cohort was restricted to Chinese adults aged ≥45 years, the generalizability of these findings to other ethnic groups or younger populations requires further investigation. In addition, an exploratory analysis suggested a smilar association with progression from T2DM to CKD; however, these findings should be considered hypothesis-generating and require validation in larger cohorts. These findings suggest that AIP‑and CumAIP-based models may be serve as practical tools for risk stratification of T2DM with CKD in middle-aged and older Chinese populations.

## Supporting information

S1 Table The association between AIP, CumAIP and T2DM with CKD using a complete case analysis (n = 5,779). (XLSX)

S2 Table Characteristics of the participants with T2DM at baseline (N = 494). (DOCX)

S3 Table AIP’s association with the risk of progression to CKD among diabetic patients in the CHARLS. (DOCX)

S4 Table CumAIP’s association with the risk of progression to CKD among diabetic patients in the CHARLS. (DOCX)

## Abbreviations

AIP: atherogenic index of plasma
CumAIP: cumulative atherogenic index of plasma
T2DM: type 2 diabetes mellitus
CKD: chronic kidney disease
TG: triglycerides
TC: total cholesterol
LDL-C: low-density lipoprotein cholesterol
HDL-C: high-density lipoprotein cholesterol
IR: insulin resistance
CHARLS: China Health and Retirement Longitudinal Study
SCr: serum creatinine
SD: standard deviation
IQR: interquartile range
OR: odds ratio
CIs: confidence intervals
ROC: receiver operating characteristic
RCS: restricted cubic splines
DKD: diabetic kidney disease
NDKD: non diabetic kidney disease
ROS: reactive oxygen species
VLDL: very-low-density lipoprotein.
