# Supplementary material for: The association between atherogenic index of plasma, cumulative atherogenic index of plasma and type 2 diabetes mellitus with chronic kidney disease among older Chinese adults: Findings from the CHARLS study
Source: PLoS One. 2026 Aug 7;21(8):e0355200. doi: 10.1371/journal.pone.0355200 (PMC13450781; doi:10.1371/journal.pone.0355200)
Supplement: Supplementary file 1 [file pone.0355200.s002.docx]

**S2 Table. Characteristics of the participants with T2DM at baseline (N = 494)**

|  | level | TOTAL | T2DM | T2DM With CKD | *P* |
| --- | --- | --- | --- | --- | --- |
| Age, years |  | 59(54,65) | 59(53,64) | 63(59,70) | ＜0.001 |
| Sex, n(%) |  |  |  |  | 0.240 |
|  | Female | 293(59.3) | 271(58.5) | 22(71.0) |  |
|  | Male | 201(40.7) | 192(41.5) | 9(29.0) |  |
| Marital, n(%) |  |  |  |  | 0.509 |
|  | Married | 440(89.1) | 414(89.4) | 26(83.9) |  |
|  | Non-married | 54(10.9) | 49(10.6) | 5(16.1) |  |
| Education, n(%) |  |  |  |  | 0.065 |
|  | College or above | 24(4.9) | 23(5.0) | 1(3.2) |  |
|  | High School | 132(26.7) | 129(27.9) | 3(9.7) |  |
|  | Primary school or below | 338(68.4) | 311(67.2) | 27(87.1) |  |
| Location, n(%) |  |  |  |  | 1.000 |
|  | City or Town | 115(23.3) | 108(23.3) | 7(22.6) |  |
|  | Village | 379(76.7) | 355(76.7) | 24(77.4) |  |
| Drinking, n(%) |  |  |  |  | 0.051 |
|  | Drink but less than once a month | 33(6.7) | 33(7.1) | 0(0.0) |  |
|  | Drink more than once a month | 87(17.6) | 85(18.4) | 2(6.5) |  |
|  | None of these | 374(75.7) | 345(74.5) | 29(93.5) |  |
| Smoking, n(%) |  |  |  |  | 0.013 |
|  | Current smoker | 116(23.5) | 110(23.8) | 6(19.4) |  |
|  | Ex-smoker | 52(10.5) | 51(11.0) | 1(3.2) |  |
|  | Non-smoker | 326(66.0) | 302(65.2) | 24(77.4) |  |
| BMI, kg/m^2^ |  | 25.38(22.86,27.79) | 25.42(22.84,27.82) | 25.08(24.11,26.73) | 0.909 |
| WC, cm |  | 91.20(84.20,98.0) | 91.20(84.20,97,7) | 92.00(85.95,99.20) | 0.152 |
| SBP, mmHg |  | 133.33(120.67,148.25) | 133.00(120.67,148.00) | 136.67(120.67,151.00) | 0.516 |
| DBP, mmHg |  | 76.83(69.33,84.67) | 77.00(68.33,84.67) | 74.67(68.17,81.33) | 0.083 |
| AIP |  | 0.13(-0.10,0.37) | 0.13(-0.11,0.37) | 0.16(-0.04,0.37) | 0.743 |
| Quartiles of AIP |  |  |  |  | 0.128 |
|  | Q1 | 124(25.1) | 121(26.1) | 3(9.7) |  |
|  | Q2 | 123(24.9) | 111(24.0) | 12(38.7) |  |
|  | Q3 | 123(24.9) | 115(24.8) | 8(25.8) |  |
|  | Q4 | 124(25.1) | 116(25.1) | 8(25.8) |  |
