# Supplementary material for: The association between atherogenic index of plasma, cumulative atherogenic index of plasma and type 2 diabetes mellitus with chronic kidney disease among older Chinese adults: Findings from the CHARLS study
Source: PLoS One. 2026 Aug 7;21(8):e0355200. doi: 10.1371/journal.pone.0355200 (PMC13450781; doi:10.1371/journal.pone.0355200)
Supplement: Supplementary file 2 [file pone.0355200.s003.docx]

**S3 Table. Association of AIP with the risk of Diabetic patients with CKD in the CHARLS**

|  | Model 1 | *p* | Model 2 | *p* | Model 3 | *p* |
| --- | --- | --- | --- | --- | --- | --- |
| AIP per IQR | 1.004[0.979,1.030] | 0.743 | 1.012[0.986,1.038] | 0.374 | 1.021[0.994,1.049] | 0.121 |
| Quartiles of AIP |  |  |  |  |  |  |
| Q1 | Ref |  | Ref |  | Ref |  |
| Q2 | 1.080[1.013,1.143] | 0.018 | 1.072[1.009,1.138] | 0.025 | 1.077[1.013,1.146] | 0.018 |
| Q3 | 1.042[0.981,1.107] | 0.190 | 1.046[0.985,1.111] | 0.140 | 1.060[0.997,1.127] | 0.063 |
| Q4 | 1.041[0.980,1.106] | 0.190 | 1.050[0.989,1.116] | 0.110 | 1.050[1.009,1.145] | 0.025 |
| *P* for trend |  | 0.355 |  | 0.199 |  | 0.050 |

Note: Model 1 represents the crude model. Model 2 was adjusted for age, sex, educational attainment, region of residence, and marital status. Model 3 was further adjusted for BMI, WC, smoking status, alcohol consumption, and blood pressure parameters.
