# Supplementary material for: The association between atherogenic index of plasma, cumulative atherogenic index of plasma and type 2 diabetes mellitus with chronic kidney disease among older Chinese adults: Findings from the CHARLS study
Source: PLoS One. 2026 Aug 7;21(8):e0355200. doi: 10.1371/journal.pone.0355200 (PMC13450781; doi:10.1371/journal.pone.0355200)
Supplement: Supplementary file 3 [file pone.0355200.s004.docx]

**S4 Table. Association of CumAIP with the risk of Diabetic patients with CKD in the CHARLS**

|  | Model 1 | *p* | Model 2 | *p* | Model 3 | *p* |
| --- | --- | --- | --- | --- | --- | --- |
| Cumulative AIP per IQR | 1.010[0.983,1.038] | 0.462 | 1.017[0.990,1.045] | 0.014 | 1.027[0.998,1.057] | 0.066 |
| Quartiles of Cum AIP |  |  |  |  |  |  |
| Q1 | Ref |  | Ref |  | Ref |  |
| Q2 | 1.050[0.989,1.078] | 0.113 | 1.007[0.988,1.114] | 0.119 | 1.052[0.990,1.117] | 0.102 |
| Q3 | 1.033[0.989,1.115] | 0.289 | 1.008[0.976,1.100] | 0.250 | 1.047[0.984,1.113] | 0.144 |
| Q4 | 1.041[0.980,1.106] | 0.192 | 1.024[0.995,1.123] | 0.075 | 1.081[1.015,1.152] | 0.016 |
| *P* for trend |  | 0.270 |  | 0.107 |  | 0.022 |

Note: Model 1 represents the crude model. Model 2 was adjusted for age, sex, educational attainment, region of residence, and marital status. Model 3 was further adjusted for BMI, WC, smoking status, alcohol consumption, and blood pressure parameters.
